# Opportunities and Challenges From Leading Trends in a Biomonitoring Project: Canadian Health Measures Survey 2007–2017

**DOI:** 10.3389/fpubh.2020.00460

**Published:** 2020-09-09

**Authors:** Yi-Sheng Chao, Chao-Jung Wu, Hsing-Chien Wu, Hui-Ting Hsu, Lien-Cheng Tsao, Yen-Po Cheng, Yi-Chun Lai, Wei-Chih Chen

**Affiliations:** ^1^Independent Researcher, Montreal, QC, Canada; ^2^Département d'informatique, Université du Québec à Montréal, Montreal, QC, Canada; ^3^Taipei Hospital, Ministry of Health and Welfare, New Taipei City, Taiwan; ^4^Changhua Christian Hospital, Changhua, Taiwan; ^5^National Yang-Ming University Hospital, Yilan, Taiwan; ^6^Attending Physician, Department of Chest Medicine, Taipei Veterans General Hospital, Taipei, Taiwan; ^7^Institute of Emergency and Critical Care Medicine, National Yang-Ming University, Taipei, Taiwan

**Keywords:** trend analysis, Canadian Health Measures Survey (CHMS), biomonitoring, time trend, body mass index (BMI)

## Abstract

**Background:** Biomonitoring can be conducted by assessing the levels of chemicals in human bodies and their surroundings, for example, as was done in the Canadian Health Measures Survey (CHMS). This study aims to report the leading increasing or decreasing biomarker trends and determine their significance.

**Methods:** We implemented a trend analysis for all variables from CHMS biomonitoring data cycles 1–5 conducted between 2007 and 2017. The associations between time and obesity were determined with linear regressions using the CHMS cycles and body mass index (BMI) as predictors.

**Results:** There were 997 unique biomarkers identified and 86 biomarkers with significant trends across cycles. Nine of the 10 leading biomarkers with the largest decreases were environmental chemicals. The levels of 1,2,3-trimethyl benzene, dodecane, palmitoleic acid, and o-xylene decreased by more than 60%. All of the 10 chemicals with the largest increases were environmental chemicals, and the levels of 1,2,4-trimethylbenzene, nonanal, and 4-methyl-2-pentanone increased by more than 200%. None of the 20 biomarkers with the largest increases or decreases between cycles were associated with BMI.

**Conclusions:** The CHMS provides the opportunity for researchers to determine associations between biomarkers and time or BMI. However, the unknown causes of trends with large magnitudes of increase or decrease and their unclear impact on Canadians' health present challenges. We recommend that the CHMS plan future cycles on leading trends and measure chemicals with both human and environmental samples.

## Background

The Canadian Health Measures Survey (CHMS) is a prominent and ongoing biomonitoring project that aims to assess the exposure and risk of environmental and non-environmental health hazards (1, 2). Both environmental chemicals in the air and water and biomarkers in blood, urine, and other bio-specimens have been obtained for quantification (3–6). Statistics Canada, the Public Health Agency of Canada, and Health Canada have been involved in the CHMS since its development before 2007 (7). The results from the CHMS help us to understand and track the levels of disease biomarkers and environmental chemicals, many of which have been considered health hazards by Health Canada (8).

One important feature of the CHMS is that it includes indoor air and tap water samples obtained at individual or household levels (1). The measurement using air and water samples enables researchers to derive nationally representative data on the levels of chemicals in their surroundings. Selected chemicals, particularly volatile organic compounds (VOCs) that include benzene and toluene, have been measured with both blood and air samples (6). In addition to the opportunities we expect in a biomonitoring project, measuring the same chemicals in blood and air samples illuminate the relationships between exposures and bio-accumulation with nationally representative data. Another opportunity for CHMS is its capacity to guide environmental and public health policies. CHMS has been used to monitor the disease burden of diabetes and hypertension in Canada (9, 10). Provincial or federal governments have enacted some policies to control exposure to environmental chemicals (11, 12). Results from the CHMS have the potential to demonstrate the effectiveness of these policies.

There are also challenges to the analysis of the CHMS data. Five reports have been published by Health Canada to describe the distribution of environmental chemicals in each of the five CHMS cycles (13–17). However, only basic statistics, including the mean, median and interquartile ranges, are reported for each biomarker (16). Occasionally, the statistics are stratified by age or sex, leading to insufficient sample sizes in certain subgroups (16). Without sufficient sample sizes, the statistics of the subgroups were not released to protect confidentiality (16).

Moreover, levels of environmental chemicals or biomarkers have not been considered for time trends. Trend analysis aims to illustrate the relationships between repeated measures (8). The time trends of many health measures have been regularly studied and updated using other biomonitoring data, especially those related to large disease burden and economic impact (18, 19). The time trend of childhood lead levels and secondhand smoke exposure have been studied to understand their respective impact on neurodevelopment and adverse health effects in the United States (20, 21). Another biomonitoring project, the German Environmental Specimen Bank (ESB), has explicitly described its objective as investigating the long-term trends of selected health hazards (22).

In contrast, trend analysis has been used only for selected chemicals from the CHMS data, such as inorganic arsenic, triclosan, bisphenol-A, and phthalates (2). The levels of certain environmental chemicals in blood, such as lead, mercury, and arsenic, have been continuously monitored by Health Canada. However, the time trends were not released in the same reports (13–17). To facilitate the trend analysis of biomonitoring data, an efficient tool to implement trend analysis for national surveys has been published recently (7).

Another challenge to the analysis of the CHMS data is the adjustment of other confounders, particularly body mass index (BMI). Controlling for BMI is important for several reasons; BMI is associated with the distributional volume of biomarkers (23). Given the same amount of chemicals, larger distributional volumes can lead to lower concentrations in human bodies (24), and BMI is an important indicator of obesity. Obesity is becoming more prevalent and has emerged as one of the major issues in public health (8, 25). It is not clear whether obesity and BMI increases may play a role in the increases or decreases of biomarker trends. The measurement of environmental chemicals and biomarkers in CHMS cycles provides opportunities to quantify the potential impact of various health hazards and assess the burden of various diseases. We aim to address the challenges to biomonitoring data analysis by conducting trend analyses for environmental chemicals and biomarkers available in CHMS data while adjusting for the CHMS cycles, a proxy measure of time, and BMI.

## Methods

We used the biomonitoring data from CHMS cycles 1–5 (8). The five cycles were implemented between 2007 and 2009, 2009 and 2011, 2012 and 2013, 2014 and 2015, and 2016 and 2017 (8). Canadians who lived on reserves, those who were institutionalized, and full-time members of the Canadian Forces made up <4% of the total population and were excluded from the sample (8). More than 5,000 Canadians residing in 10 provinces aged 3–79 years were interviewed in each cycle (8). There were face-to-face interviews to record demographic and socioeconomic characteristics, as well as mobile clinic visits to retrieve blood and urine samples (8). Data on accelerometer-measured daily activities, lung function measured by a spirometer, medication, diagnosis of selected major disease, lifestyle, and health behavior, such as smoking and alcohol consumption, were also obtained (8).

### Biomarker List and Variable Search

A comprehensive list of 447 unique biomarkers was provided in the CHMS cycles 1–8 Content Summary, 390 of which were available in cycles 1–5. There were 13 major themes in the biomarkers that are described in the Content Summary. These themes are allergies, bone health, cardiovascular health, chemistry panel, complete blood count, diabetes, environmental exposure, general characterization, infection markers, kidney health, nutritional status, reproductive hormones, and thyroid status (6). The environmental exposure biomarkers or environmental chemicals included measurements that quantified the levels of chemicals in blood, urine, air, or tap water (5). Indoor air samples were determined at both personal and household levels (5). Non-environmental biomarkers were measured with either blood or urine samples in cycles 1–5.

The variable names of the biomarkers listed in the Content Summary (6) were not provided in the list. Therefore, it was necessary to match the biomarkers in the Content Summary list with the variables in the CHMS data dictionaries. To do this, all variable names were extracted from cycles 1–5 data dictionaries that were available from Statistics Canada. All CHMS variables available in the data dictionaries were screened to match the biomarker names in the Content Summary. In addition to the biomarkers listed in the Content Summary (6), the measures of vital signs including resting blood pressure (26), respiratory rates and heart rates were also included for analysis under the cardiovascular health theme (27). Among the 447 unique biomarkers or environmental chemicals in the Content Summary, 107 could not be linked to the CHMS variables. This led to the identification of 997 variables that were potential environmental or non-environmental biomarkers or environmental chemicals in the CHMS data sets, including administrative variables with names similar to the biomarkers or environmental chemicals, which only provided information on the limits of biomarker detection levels.

### Variable Processing and Derived Variables

The trend analysis of all CHMS variables with and without adjusting for covariates was implemented. There were 54,235 variables in 85 data files from cycles 1–5 released before September 2019 (8). There were 16,727 variables in 35 files related to the use of bootstrap weights. The essential variables to control for survey design included sites, regions, CHMS cycles, and bootstrap weights. These variables were identified and introduced into the descriptive analysis and regression models. Unfortunately, there were discrepancies in the variable definitions across the CHMS cycles that needed to be resolved. For example, the levels of glucose and vitamin D were quantified with serum or plasma. Due to the limited differences between plasma and serum levels for respective biomarkers (8), the variables representing glucose or vitamin D levels were respectively unified. The glucose levels were measured among fasted individuals in cycles 3 and 4 and non-fasting subjects in cycles 1 and 2. To resolve these discrepancies, the glucose levels from cycles 3 and 4 were then labeled as fasting samples and analyzed separately from those from the non-fasting subjects in cycles 1 and 2. The levels of fibrinogen were measured with different units, g/cL in cycle 1 and dg/L in cycle 2, and to correct for this issue, they were converted to g/L.

### Data Cleaning and Editing

The values representing “not applicable,” “don't know,” “not stated,” and “not applicable” in all variables were recoded to missing (8). For certain measures, there were ranges of detection, and the levels of biomarkers or environmental chemicals were measurable only within these ranges. The ranges of detection might be documented in the data dictionaries or stored in separate variables. The values below the lower limits of the detection levels were replaced with half of the lower limits of detection levels, according to the imputation methods adopted by Health Canada (8, 13–16). The values representing measures higher than the upper limits of detection were replaced with 110% of the maximal levels of detection. Using the standard adopted by Health Canada, if more than 40% of the unweighted samples were found to have measures lower or higher than the limits of detection, this variable was not used for descriptive or trend analysis across cycles (8).

### Descriptive Analysis and the Association With BMI

The basic statistics of all CHMS variables were gathered while controlling for the sampling frames and survey design (8). The statistics, including the mean and 95% confidence intervals (CIs), quartiles, and weighted sample sizes, were documented. For repeated measures, the rates of increase or decrease compared to the baseline were annualized based on the number of cycles since the first measures. For example, the levels of urine 2-hydroxychrysene, a type of chrysenes or environmental chemical, were measured in cycles 2 and 3. The levels in cycle 2 were used as the baseline. The annualized rate of increase or decrease was the geometric mean of the change rates between cycles (28). For example, the levels of biomarker A might increase up to 150% from cycles 1 to 4. The annualized increase rate was calculated as the cube root of 1.5.

For the continuous variables, the relationship with covariates was determined using multiple linear regressions while controlling for survey design (8). For binominal biomarker variables that only included two types of responses, their relationship with BMI was analyzed with multiple logistic regressions. In addition to BMI, there were other candidate predictors: sex, age, household income, education, CHMS cycles, use of over-the-counter drugs, and prescription use (8). The minimum and maximum ages of participants were 3 and 79 years, respectively (8). Household income was reported in Canadian dollars (8). Educational attainment included four categories that represented the highest levels of education that participants had attained: less than secondary school graduation, secondary school graduation, some post-secondary education, and post-secondary graduation (8). To avoid overlap with the trend analysis mentioned below, the CHMS cycles were converted to dummy variables. The first CHMS cycle, in which the dependent variables were measured, was used as the baseline. If the variables were repeatedly measured, the fixed effects of subsequent cycles were controlled for.

For certain variables, the predictors might not be used because only one of the categories of the predictors applied to the related outcomes in these cases. The predictors were dropped from the regression. For example, only males were eligible for a question asking if they had prostate cancer, and sex was not used as a predictor for the diagnosis of prostate cancer. For variables that were only applicable to school-aged children, education was not used as a predictor. The levels of total arsenic in blood were only measured in cycle 1, and the CHMS cycle was not used as a predictor. Collinearity was assessed with variance inflation factors (29). If the squared variance inflation factors were greater than two, the predictors were documented and reviewed for collinearity.

There was a minimum sample size requirement for the regression analysis to control for survey design and to protect the confidentiality of the respondents (4). There should be a least one subject in each sampling site in each cycle. In total, there should be at least 100 observations for each cycle to meet the minimum sample size requirement.

### Trend Analysis

Trends in CHMS variables were analyzed as the mean and 95% CIs across the CHMS cycles (8). The objective of the trend analysis was to understand whether there was an upward or downward trend across time or cycles for each variable (8). The CHMS cycles measured on a continuous scale were used as a proxy for time and the only predictor (8). If the variables were ever repeated and there were sufficient sample sizes for all cycles—the variables were eligible for trend analysis (8). The ratios of the repeated measures defined the upward or downward trend means compared to baseline levels, the first measurement in the CHMS. When the 95% CIs of the ratios in subsequent cycles included one, there were no significant increasing or decreasing trends.

### Control for Survey Design

Stratified sampling was adopted in the CHMS and should be controlled for to obtain national estimates (8). To meet the requirement for degrees of freedom, the variables related to survey design were identified for all variables, including weights, CHMS cycles, interview sites, provinces of residence, and bootstrap weights (8). The results in this study are all weighted statistics. Two-tailed *p* < 0.05 were considered statistically significant. All statistical analyses were conducted with R (v 3.20) (30) and RStudio (v 0.98.1103) (31).

## Results

There were 997 biomarkers or environmental chemicals identified by matching the variables listed in the Content Summary and the Data Dictionaries. Population characteristics are listed in Table 1. There were more than 29 million Canadians represented in each of the five cycles, and the population numbers increased by cycle. About half of Canadians were female in the five cycles. Household income increased continuously. The mean age and BMI, respectively, decreased and increased across the CHMS cycles. On average, BMI increased at a rate of 1.002 per cycle.

**Table 1:** Characteristics of Canadians in the Canadian Health Measures Survey cycles 1–5.

**Cycles**	**Cycle 1**	**Cycle 2**	**Cycle 3**	**Cycle 4**	**Cycle 5**
Study time	2007–2009	2009 and 2011	2011–2013	2014–2015	2016–2017
Unweighted sample sizes (*n*)	5,604	6,395	5,785	5,794	5,786
Number of variables	6,798	6,971	9,709	7,686	2,928
Number of repeated measures	0	2,448	3,412	5,576	1,625
Number of biomarkers	327	925	864	327	143
Number of variables used to provide limits of detection or quantification (administrative)	0	168	176	0	0
Weighted *N* with full weights	29,235,444	31,026,646	31,663,898	32,275,596	32,255,596
Proportions of females	0.502	0.501	0.501	0.501	0.501
Mean ages (years)	39.3	38.6	39	39.3	39.4
Mean BMI	26	25.8	25.9	26.2	26.3
Mean household income (Canadian dollars)	77,818.5	80,085.7	84,779.2	92,165.8	93,065.4

*BMI, body mass index*.

In summary, 808 CHMS variables and 103 biomarkers or environmental chemicals repeatedly measured were significantly associated with the CHMS cycles on a continuous scale. Of all the variables, 1,147 variables and 167 biomarkers were significantly associated with BMI after controlling for the CHMS cycles, age, sex, household income, and education. If the use of prescription medication was also controlled, there were 440 variables and 53 biomarkers that were significantly associated with BMI. For biomarkers or environmental chemicals, there were 65, three, and one variable that increased by more than 10% compared to the levels measured one, two, and three cycles ago, respectively. There were 80, seven, and three biomarker variables that decreased by more than 10% compared to the levels measured one, two, and three cycles ago, respectively. Most of the biomarkers that increased or decreased at high rates were environmental chemicals.

**Figure 1:**
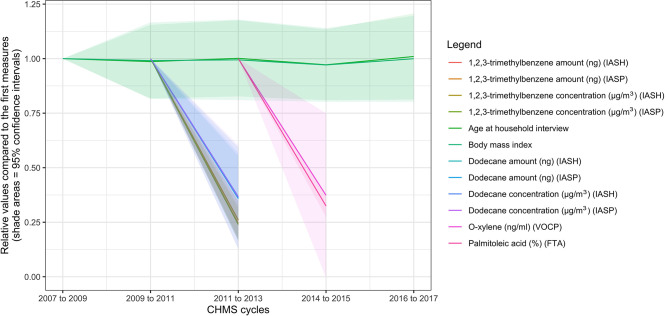
Ten biomarkers with the highest rates of decrease in the biomonitoring data from the Canadian Health Measures Survey. FTA, red blood cell fatty acids subsample; IASH, indoor air household level; IASP, indoor air person level; VOCP, volatile organic compounds person level.

**Figure 2:**
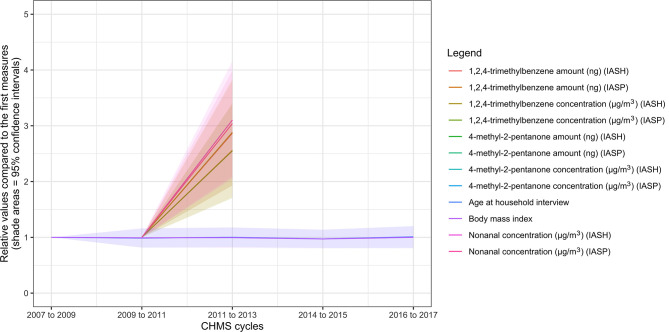
Ten biomarkers with the highest rates of increase in the biomonitoring data from the Canadian Health Measures Survey. IASH, indoor air household level; IASP, indoor air person level.

To demonstrate the results, the leading trends of decrease and increase were plotted in Figures 1, 2, compared to the trends of age in years and BMI in kg/m^2^. Nine of the 10 variables that decreased the most rapidly were environmental chemicals. These variables are related to three chemicals and one biomarker in Figure 1: 1,2,3-trimethylbenzene (amount in ng or concentration in μg/m^3^, air samples), dodecane (amount in ng or concentration in μg/m^3^, air samples), palmitoleic acid (%, blood samples), and o-xylene (ng/ml, air samples). Only palmitoleic acid proportions (blood samples) decreased by more than 60% between cycles 2 and 3 and was not an environmental chemical. The amounts or concentrations of 1,2,3-trimethylbenzene, and dodecane were measured at the household and personal levels. The ratios of the four measures of 1,2,3-trimethylbenzene were all around 0.25 in cycle 3 compared with cycle 2, as shown in Figure 1 (see Appendix 1 for details). The ratios of the four measures of dodecane were around 0.37 in cycle 3, compared to cycle 2. The ratio of palmitoleic acid (in proportions) was 0.34 (95% CI = 0.29–0.40) in cycle 4, compared to cycle 3. O-xylene, a type of volatile organic compounds (32), was measured for the concentrations, and the ratio was 0.37 (95% CI = −0.003 to 0.75) in cycle 3, compared to cycle 2. Because most environmental chemicals were measured only in cycles 2 and 3, there was no information on these environmental chemicals in cycles 1 and 4. Palmitoleic acid (%) was considered a cardiovascular health marker (33) and the ratio was 0.32 (95% CI = 0.27–0.37) in cycle 4, compared with cycle 3. None of the levels of the variables in Figure 1 were related to BMI in the regression models (*p* > 0.05 for all).

The variables, which increased the most rapidly, are shown in Figure 2 (see Appendix 2 for details). The 10 leading variables were all environmental chemicals: 1,2,4-trimethylbenzene (amount in ng or concentration in μg/m^3^, air samples), nonanal (air samples), and 4-methyl-2-pentanone (amount in ng or concentration in μg/m^3^, air samples). The ratios of 4-methyl-2-pentanone concentrations were 6.08 (95% CI = 2.18–9.97) and 6.13 (95% CI = 2.62–9.64) at personal and household levels respectively in cycle 3, compared to cycle 2. The ratios of 4-methyl-2-pentanone amounts were 5.29 (95% CI = 1.86–8.71) and 5.32 (95% CI = 2.12–8.51) at personal and household levels respectively in cycle 3, compared to cycle 2. The ratios of nonanal levels were 3.10 (95% CI = 2.05–4.15) and 3.04 (95% CI = 2.09–3.99) at personal and household levels, respectively, in cycle 3, compared to cycle 2. The ratios of the 1,2,4-trimethylbenzene concentrations or amount at the personal or household levels were between 2.55 and 2.89 in cycle 3, compared to cycle 2. None of the levels of the variables in Figure 2 were significantly associated with BMI (*p* > 0.05 for all).

## Discussion

The CHMS biomonitoring project provides opportunities for researchers and policymakers to understand the exposure to health hazards and potential disease burden based on environmental chemicals and disease biomarkers, respectively. We have identified 997 biomarker-related variables in cycle 1–5, and there will be new biomarkers measured in subsequent cycles. However, there are also challenges to the analysis of the CHMS data, especially regarding the investigation of time trends and biomarkers' or environmental chemicals' associations with other confounders. First, a comprehensive study of all CHMS biomarkers is needed. If this is not possible, high-priority biomarkers or environmental chemicals for analysis need to be decided. The leading increasing or decreasing trends we identified have not been well-studied by researchers or CHMS administrators to our knowledge. Although there are a large number of variables collected in the CHMS, the number of variables in the CHMS remains manageable because efficient tools for trend analysis have been developed (8, 25). We urge the CHMS administrators to introduce these tools and release the latest trends of all environmental chemicals and biomarkers.

Second, the interpretation of the trends requires in-depth knowledge and multi-disciplinary expertise. The environmental and non-environmental biomarkers are distinctive and have different characteristics. Three environmental chemicals in this study have the highest rates of decrease: 1,2,3-trimethylbenzene, dodecane, o-xylene (above measured with air samples), and palmitoleic acid (measured with blood samples). 1,2,3-trimethylbenzene can cause skin irritations and other symptoms (34). Dodecane can be used as a solvent for industrial use (35). O-xylene, a flammable oily liquid, is used to produce other chemicals or drugs (36). Palmitoleic acid is associated with metabolic risks in mixed directions (37).

Three environmental chemicals measured with air samples show the highest rates of increase: 1,2,4-trimethylbenzene, nonanal, and 4-methyl-2-pentanone. 1,2,4-trimethylbenzene is an important gasoline additive and is often used in the petroleum industry (38). Nonanal can be derived from nonanoic acid, and its metabolites have been observed in cancer metabolism (39). 4-methyl-2-pentanone, a type of colorless ketone, is an important industrial solvent (40). These chemicals and biomarkers are produced from different sources with varying degrees of health impact, although the potential sources and the overall impact remain unclear. Experts from several disciplines will be needed to further understand the sources and effects of these chemicals.

Third, there are other covariates to be investigated. We first focused on demographic characteristics and found that the role of BMI on the leading increasing or decreasing trends seems to be limited. The increase rate of BMI is relatively low, 0.2% per cycle on average (see Appendix 1 for details), and many biomarkers increased at rates much higher than those of BMI. The role of diet and other factors related to exposure to environmental chemicals will need to be investigated.

Fourth, policy evaluation should be implemented immediately for these leading trends. Air quality has been monitored, and federal or provincial governments in Canada have revised standards (41). For example, Ontario, Canada's most populated province, has established air quality standards for 1,2,3-Trimethylbenzene and 1,2,4-Trimethylbenzene, two of the leading trends identified (11). In 2019, a risk management plan for 4-methyl-2-pentanone was proposed by the federal government (12). At this time, there has not been any progress published (12). Immediate action is required to assess the risks of these leading trends, and policy evaluation is warranted.

Lastly, the CHMS has the potential to be used to understand the relationships between exposure and accumulation in human bodies. However, only selected chemicals were measured with both blood and air samples for the same individuals, such as benzene in the CHMS cycle 2. None of the environmental chemicals with the leading trends we identified were measured with both blood and air samples in cycle 2 or 3. If the CHMS plans remain the same, there will be more biomarkers available for trend analysis and the comparison between exposure (air or water samples) and accumulation (blood samples) in the CHMS cycle 6 (6).

### Recommendations for the CHMS Biomonitoring

After our extensive review of the codebook and dictionaries, we have several recommendations for the CHMS for biomonitoring. First, the identification of biomarkers in publicly accessible documents can be improved and made consistent. For example, several of the biomarkers or environmental chemicals listed in the content summary could not be retrieved in the data dictionaries. These biomarkers include 1-hydroxynaphthol and 2-hydroxynaphthol measured in urine, tribromomethane, and trichloromethane measured with air samples (6). Second, some basic clinical measures do not seem to be considered, such as blood osmolality and urine sodium that are important for kidney function evaluation (42). For the research community, it remains unclear why some measures were included while others were not. Our results show that biomarkers or environmental chemicals with large fluctuations are not necessarily reviewed or measured more frequently.

Third, the immediate implementation of a trend analysis of the CHMS data has several benefits. Trend analysis is a tool that can be used to identify some of the data errors (8, 25). Inconsistency in the measurement unit of fibrinogen, g/L and g/dL, is related to a 10-fold change that is much higher than the rate increases of any other variables and can be easily identified with trend analysis (personal communications with Statistics Canada). The identification of rapidly increasing or decreasing environmental chemicals is one of the first steps to assess the risks to human health. Understanding the significance of time trends is vital to plan for the biomarkers or environmental chemicals in subsequent biomonitoring. Currently, the biomarkers or environmental chemicals for biomonitoring have been decided up to CHMS cycle 8. The rapidly decreasing or increasing biomarkers identified in the study will not be measured in CHMS cycle 6 (6). The results of trend analysis may help prioritize the biomarkers or environmental chemicals for monitoring.

Lastly, the overall direction of CHMS can be better aligned to certain contexts. For example, if oriented to mimic the primary care settings, the CHMS can introduce other routine measures in primary care, such as body temperature and frequencies of common conditions, including common colds and influenza infections. Currently, there are more than 60 cardiovascular health variables conducted or planned for the CHMS. If oriented for the largest disease burden, cardiovascular disease (43), the CHMS can adopt measures in mean arterial pressure or even electrocardiography in the future.

### Strengths and Limitations

The strengths of this study include the national representativeness of the sample, repeated measures of the majority of variables, well-structured data, and the interpretability of the results. However, there are several limitations to the trend analysis. First, data processing can be improved. Health Canada imputes the values below the limits lower than the detection limits by assigning half of the detection limits (14, 15, 17). There are other advanced methods available to impute censored information (44). Second, the programming codes are written at the Research Data Center under time and physical constraints and could be further streamlined for simplicity and execution efficiency. Four-day computation time may be further reduced (8). Third, the rapidly increasing or decreasing trends may be due to reasons other than the changes in their distributions or concentrations over time. Factors, such as sampling frames, non-response, sampling errors, measurement errors, and changes in measurement standards or methods, may play a role in the biomarker levels over time.

## Conclusion

Trend analysis is a highly feasible method to screen all CHMS variables and can be used to select biomarkers increasing or decreasing rapidly across cycles. The associations with time and BMI are also possible to test. This helps to assess the potential health consequences related to the biomarkers or environmental chemicals and prioritize the biomarkers or environmental chemicals for investigation. We recommend the CHMS to plan future cycles based on the results of trend analysis, especially those increasing or decreasing at high rates. It is also possible to extend this trend analysis framework to other similar Statistics Canada data products, especially those with missing values coded in the same way as they are in the CHMS.

## Data Availability Statement

The datasets used for this study will not be made publicly available. It is against the Statistics Act of Canada to release the CHMS data or identify the individuals participating in the CHMS. Requests can be directed to Statistics Canada, statcan.maddli-damidd.statcan@canada.ca.

## Ethics Statement

This secondary data analysis was approved by the ethics review committee at the Center Hospitalier de l'Université de Montréal. All methods were performed in accordance with the guidelines and regulations relevant to the analysis of public data. Written informed consent for the Canadian Health Measures Survey was obtained by Statistics Canada and not accessible to researchers.

## Consent for Publication

Participants' consent for publication is not required for this data analysis project.

## Author Contributions

Y-SC conceptualized and designed this study, managed and analyzed data, and drafted the manuscript. C-JW assisted in data management and computation. H-CW, H-TH, L-CT, Y-PC, Y-CL, and W-CC participated in the design of this study. All authors reviewed and approved the manuscript.

## Conflict of Interest

Y-SC is currently employed by the Canadian Agency for Drugs and Technologies in Health. The remaining authors declare that the research was conducted in the absence of any commercial or financial relationships that could be construed as a potential conflict of interest.

## Abbreviations

CHMS: Canadian Health Measures Survey
BMI: body mass index.

## References

[1] Haines DA Saravanabhavan G Werry K Khoury C. An overview of human biomonitoring of environmental chemicals in the Canadian Health Measures Survey: 2007-2019. Int J Hygiene Environ Health. (2017) 220(2 Pt A):13–28. 10.1016/j.ijheh.2016.08.002 27601095

[2] St-Amand A Werry K Aylward LL Hays SM Nong A. Screening of population level biomonitoring data from the Canadian Health Measures Survey in a risk-based context. Toxicol Lett. (2014) 231:126–34. 10.1016/j.toxlet.2014.10.019 25455445

[3] Day B Langlois R Tremblay M Knoppers BM. Canadian Health Measures Survey: ethical, legal and social issues. Health Rep. (2007) 18(Suppl):37–51. 18210869

[4] Giroux S. Canadian Health Measures Survey: sampling strategy overview. Health Rep. (2007) 18(Suppl):31–6. 18210868

[5] Haines DA Murray J. Human biomonitoring of environmental chemicals-Early results of the 2007-2009 Canadian Health Measures Survey for males and females. Int J Hygiene Environ Health. (2012) 215:133–7. 10.1016/j.ijheh.2011.09.008 22001329

[6] Statistics Canada Canadian Health Measures Survey (CHMS) Content Summary for Cycles 1 to 8. In: Statistics Canada, editor. Ottawa, ON: Statistics Canada (2015).

[7] Committee to Review the Department of the Interior's Biomonitoring of Environmental Status and Trends Program Board on Environmental Studies and Toxicology A Review of the Biomonitoring of Environmental Status and Trends Program: The Draft Detailed Plan. Committee to Review the Department of the Interior's Biomonitoring of Environmental Status and Trends Program, editor. Washington, DC: National Academy Press (1995).

[8] Chao Y-S Wu C-J Wu H-C Chen W-C. Trend analysis for national surveys: application to all variables from the Canadian Health Measures Survey cycle 1 to 4. PLoS One. (2018) 13:e0200127. 10.1371/journal.pone.0200127 30092046 PMC6084849

[9] Rosella LC Lebenbaum M Fitzpatrick T Zuk A Booth GL. Prevalence of prediabetes and undiagnosed diabetes in Canada (2007-2011) according to fasting plasma glucose and HbA1c screening criteria. Diabetes Care. (2015) 38:1299–305. 10.2337/dc14-2474 25852207

[10] McAlister FA Wilkins K Joffres M Leenen FH Fodor G Gee M. Changes in the rates of awareness, treatment and control of hypertension in Canada over the past two decades. CMAJ. (2011) 183:1007–13. 10.1503/cmaj.101767 21576297 PMC3114892

[11] Ontario Ministry of the Environment Ontario Air Standards For Trimethylbenzenes: 1,2,3-Trimethylbenzene 1,2,4-Trimethylbenzene 1,3,5-Trimethylbenzene. Toronto, ON: Ontario Ministry of the Environment (2007). Available online at: https://collections.ola.org/mon/20000/277838.pdf.

[12] Environment and Climate Change Canada Health Canada Risk Management Scope for Ketones Specifically 2-Butanone (MEK); 4-Methyl-2-Pentanone (MIBK); 2,4-Pentanedione (2,4-PD). Ottawa, ON: Government of Canada (2019). Available online at: https://www.canada.ca/en/environment-climate-change/services/evaluating-existing-substances/risk-management-scope-ketones-butanone-methyl-pentanone-pentanedione.html.

[13] Health Canada Second Report on Human Biomonitoring of Environmental Chemicals in Canada. Ottawa, ON: Health Canada (2013).

[14] Health Canada Report on Human Biomonitoring of Environmental Chemicals in Canada. Ottawa, ON: Health Canada (2010). Available online at: http://www.hc-sc.gc.ca/ewh-semt/alt_formats/hecs-sesc/pdf/pubs/contaminants/chms-ecms/report-rapport-eng.pdf.

[15] Health Canada Third Report on Human Biomonitoring of Environmental Chemicals in Canada. Ottawa, ON: Health Canada (2015). Available online at: http://www.hc-sc.gc.ca/ewh-semt/alt_formats/pdf/pubs/contaminants/chms-ecms-cycle3/chms-ecms-cycle3-eng.pdf.

[16] Health Canada. Fourth Report on Human Biomonitoring of Environmental Chemicals in Canada. Ottawa, ON: Health Canada (2017). Available online at: https://www.canada.ca/content/dam/hc-sc/documents/services/environmental-workplace-health/reports-publications/environmental-contaminants/fourth-report-human-biomonitoring-environmental-chemicals-canada/fourth-report-human-biomonitoring-environmental-chemicals-canada-eng.pdf.

[17] Health Canada Second Report on Human Biomonitoring of Environmental Chemicals in Canada. Health Canada, editor. Ottawa, ON: Health Canada (2013).

[18] Dombrovskiy VY Martin AA Sunderram J Paz HL. Rapid increase in hospitalization and mortality rates for severe sepsis in the United States: a trend analysis from 1993 to 2003. Critical Care Med. (2007) 35:1244–50. 10.1097/01.CCM.0000261890.41311.E9 17414736

[19] Sturm R. Increases in morbid obesity in the USA: 2000-2005. Public health. (2007) 121:492–6. 10.1016/j.puhe.2007.01.006 17399752 PMC2864630

[20] Raymond J. Blood lead levels in children aged <5 years-United States, 2007-2013. MMWR Morb Mortal Wkly Rep. (2016) 63:66–72. 10.15585/mmwr.mm6355a6 27736835

[21] Pirkle JL Bernert JT Caudill SP Sosnoff CS Pechacek TF. Trends in the exposure of nonsmokers in the US population to secondhand smoke: 1988-2002. Environ Health Perspect. (2006) 114:853–8. 10.1289/ehp.8850 16759984 PMC1480505

[22] Göen T Lermen D Hildebrand J Bartel-Steinbach M Weber T Kolossa-Gehring M. Discovering time-trends of the German populations exposure to contaminants by analysis of human samples of the German Environmental Specimen Bank (ESB). Toxicol Lett. (2018) 298:194–200. 10.1016/j.toxlet.2018.06.007 29906498

[23] Chao YS Brunel L Faris P Veugelers PJ. The importance of dose, frequency and duration of vitamin d supplementation for plasma 25-hydroxyvitamin d. Nutrients. (2013) 5:4067–78. 10.3390/nu5104067 24152747 PMC3820059

[24] Institute of Medicine (US) Committee to Review Dietary Reference Intakes for Vitamin D and Calcium; Ross AC Taylor CL Yaktine AL Del Valle HB editors Dietary Reference Intakes for Calcium and Vitamin D. Washington, DC: The National Academies Press (2011). 21796828

[25] Chao Y-S Wu C-J Wu H-C Chen W-C. Drug trends among non-institutionalized Canadians and the impact of data collection changes in the Canadian Health Measures Survey 2007 to 2015. PLoS One. (2019) 14:e0214718. 10.1371/journal.pone.0214718 30978234 PMC6461261

[26] Bryan S Saint-Pierre LM Campbell N Clarke J Tremblay MS. Resting blood pressure and heart rate measurement in the Canadian Health Measures Survey, cycle 1. Health Rep. (2010) 21:71–8. 20426229

[27] Heger JW Niemann JT Criley JM Cardiology. 4th ed. Baltimore: Lippincott Williams and Wilkins (2004).

[28] Lewis M Applied Statistics for Economists. Philadelphia, PA: Taylor & Francis (2012).

[29] Fox J Friendly GG Graves S Heiberger R Monette G Nilsson H The Car Package. Vienna, Austria: R Foundation for Statistical Computing (2007).

[30] R Development Core Team R: A Language and Environment for Statistical Computing. Vienna, Austria: R Foundation for Statistical Computing (2016).

[31] RStudio Team RStudio: Integrated Development for R. Boston, MA: RStudio, Inc (2016).

[32] Statistics Canada Canadian Health Measures Survey: Indoor Air Volatile Organic Compound Data, 2014 and 2015. Ottawa, ON: Statistics Canada (2017). Available online at: https://www150.statcan.gc.ca/n1/daily-quotidien/171031/dq171031f-eng.htm.

[33] Hernandez EM Chapter 4: Specialty Oils: Functional and Nutraceutical Properties. In: Sanders TAB, editor. Functional Dietary Lipids. Sawston; Cambridge, UK: Woodhead Publishing (2016). p. 69–101.

[34] Bernabei M Reda R Galiero R Bocchinfuso G. Determination of total and polycyclic aromatic hydrocarbons in aviation jet fuel. J Chromatogr A. (2003) 985:197–203. 10.1016/S0021-9673(02)01826-5 12580487

[35] Rydberg J Solvent *Extraction Principles and Practice, Revised and Expanded*. Oxfordshire: Taylor & Francis (2004).

[36] Kandyala R Raghavendra SPC Rajasekharan ST. Xylene: an overview of its health hazards and preventive measures. J Oral Maxillofac Pathol. (2010) 14:1. 10.4103/0973-029X.64299 21180450 PMC2996004

[37] Siscovick DS Hotamisligil GS Cao H King IB Lemaitre RN Song X. Circulating palmitoleic acid and risk of metabolic abnormalities and new-onset diabetes. Am J Clin Nutr. (2010) 92:1350–8. 10.3945/ajcn.110.003970 20943795 PMC2980960

[38] Delaware Health Social Services 1,2,4-TRIMETHYLBENZENE. New Castle, DE: Delaware Health and Social Services (2013). Available online at: https://www.dhss.delaware.gov/dph/files/tmb124faq.pdf.

[39] National Center for Biotechnology Information PubChem Compound Database; CID=31289. Available online at: https://pubchem.ncbi.nlm.nih.gov/compound/31289.

[40] Sifniades S Levy AB Bahl H Acetone. In: Ullmann's Encyclopedia of Industrial Chemistry. New York, NY: Wiley (2011).

[41] McKitrick R The politics of pollution: party regimes and air quality in Canada. Can J Econ. (2006) 39:604–20. 10.1111/j.0008-4085.2006.00362.x

[42] Longo D Fauci A Kasper D Hauser S Jameson J Loscalzo J Harrison's Principles of Internal Medicine. 19th ed. McGraw-Hill Education (2015).

[43] Murray CJ Lopez AD. Alternative projections of mortality and disability by cause 1990-2020: Global Burden of Disease Study. Lancet. (1997) 349:1498–504. 10.1016/S0140-6736(96)07492-2 9167458

[44] Puhani P The Heckman correction for sample selection and its critique. J Econ Surv. (2000) 14:53–68 10.1111/1467-6419.00104

